# A Nephrologist’s Dilemma: Severe Granulomatous Acute Interstitial Nephritis (AIN) and Pancreatitis From Allopurinol‑Induced Drug Reaction With Eosinophilia and Systemic Symptoms (DRESS)

**DOI:** 10.7759/cureus.110797

**Published:** 2026-06-13

**Authors:** Said Al Zein

**Affiliations:** 1 School of Medicine, University of Pittsburgh, Williamsport, USA

**Keywords:** acute interstitial nephritis, acute pancreatitis, allopurinol hypersensitivity, dress syndrome, drug-induced hypersensitivity, eosinophilia, granulomatous interstitial nephritis, renal biopsy

## Abstract

Allopurinol is a well‑recognized trigger of drug reaction with eosinophilia and systemic symptoms (DRESS), but presentation with both severe granulomatous interstitial nephritis and acute pancreatitis is uncommon and diagnostically challenging. A 71‑year‑old man recently started on allopurinol presented with fever, diffuse rash, altered mental status, and rapidly progressive acute kidney injury (AKI). His course was complicated by marked eosinophilia, hepatocellular injury, and severe pancreatitis. Infectious and autoimmune evaluations were unrevealing. Renal biopsy demonstrated granulomatous interstitial nephritis with abundant eosinophils, confirming a drug‑induced hypersensitivity reaction. High‑dose corticosteroids led to improvement in mental status, resolution of rash with desquamation, and gradual recovery of kidney function. This case highlights the diagnostic dilemma posed by multisystem involvement in DRESS and underscores the pivotal role of renal biopsy in establishing the etiology of severe AKI in complex presentations.

## Introduction

Drug reaction with eosinophilia and systemic symptoms (DRESS) is a severe, delayed hypersensitivity reaction characterized by fever, diffuse rash, hematologic abnormalities (eosinophilia and/or atypical lymphocytosis), and multiorgan involvement, typically appearing two to six weeks after initiation of the culprit drug [1]. Mortality ranges from 3.8% to 10%, driven primarily by hepatic failure or severe multiorgan dysfunction [1]. Allopurinol remains one of the most frequent triggers, particularly in patients with chronic kidney disease (CKD) [2]. Renal involvement occurs in up to 40% of DRESS cases and is most often acute interstitial nephritis (AIN); granulomatous interstitial nephritis is a distinctly uncommon histologic variant that can further obscure the diagnosis when systemic features mimic infection, pancreatitis, or autoimmune disease [3]. Pancreatic involvement is rare (<5% of cases) but documented, usually as acute pancreatitis within the systemic inflammatory cascade [4]. The presence of pancreatitis, altered mental status, fever, and progressive acute kidney injury (AKI) can mimic infectious, metabolic, or autoimmune conditions, delaying recognition of the underlying drug reaction.

In presentations with overlapping AKI etiologies (volume depletion, contrast exposure, and systemic inflammation), renal biopsy is essential to confirm drug-induced hypersensitivity and to direct timely immunosuppression. We present a case of allopurinol-induced DRESS featuring biopsy-proven granulomatous AIN and concurrent acute pancreatitis, highlighting the diagnostic dilemma faced by the nephrologist in multisystem drug reactions.

## Case presentation

A 71-year-old man with hypertension, CKD (baseline creatinine: 1.6 mg/dL), and gout presented with four days of fever, diffuse pruritic rash, generalized weakness, and abdominal discomfort. Three weeks before the presentation, he had been started on allopurinol 100 mg daily for gout. He denied alcohol use, recent travel, sick contacts, or any other new medications.

On presentation, he was febrile (38.6°C) and ill-appearing, with a diffuse morbilliform rash involving the trunk and extremities without mucosal involvement. Initial laboratory studies demonstrated AKI (creatinine: 2.4 mg/dL), leukocytosis, eosinophilia, elevated liver enzymes, and lipase of 1,940 U/L. Urinalysis showed 36 WBC/hpf, 63 RBC/hpf, granular casts, +1 protein on dipstick, and urine protein-to-creatinine ratio of 1 g/g. Computed tomography of the abdomen with intravenous contrast showed peripancreatic fat stranding consistent with acute pancreatitis without evidence of gallstones or biliary obstruction (Figure 1). Infectious evaluation, including blood cultures, hepatitis panel, HIV, Epstein-Barr virus, cytomegalovirus, and tick-borne disease testing, was unrevealing.

**Figure 1:**
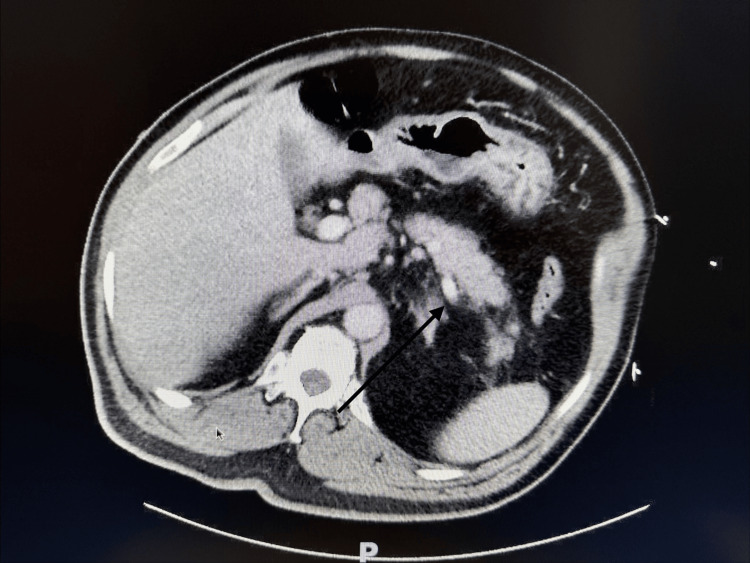
CT abdomen demonstrating acute pancreatitis Contrast-enhanced CT abdomen showing peripancreatic fat stranding consistent with acute pancreatitis. The arrow indicates the pancreas.

Given the patient's presentation with acute pancreatitis and fever, the initial diagnostic focus centered on determining the etiology of pancreatitis. Empiric vancomycin and cefepime were administered while infectious causes were investigated. The AKI was initially attributed to a combination of systemic illness, pancreatitis-associated volume depletion, and subsequent intravenous contrast exposure.

Over the following several days, the patient's rash progressed, eosinophilia increased, and kidney function continued to deteriorate despite supportive care and intravenous fluids. Dermatology evaluation favored a drug eruption. Concurrently, the patient developed worsening mental status, becoming progressively somnolent though remaining arousable. CT of the head showed no acute infarct or hemorrhage, with chronic small vessel white matter disease and volume loss; the absence of an acute structural process supported a toxic-metabolic and inflammatory etiology for the encephalopathy. The combination of fever, diffuse rash, eosinophilia, hepatitis, pancreatitis, and progressive AKI raised concern for DRESS syndrome. Human herpesvirus 6 (HHV-6) polymerase chain reaction subsequently returned positive, while autoimmune serologies, including ANA, anti-dsDNA, ANCA, and cryoglobulins, were negative, and complement levels were normal.

Despite discontinuation of allopurinol upon admission, the patient's creatinine continued to rise, increasing from 2.4 mg/dL on admission to 4.1 mg/dL by hospital day 4. Given the rapidly progressive AKI and the presence of several competing potential etiologies, including pancreatitis-associated kidney injury, intravenous contrast exposure, systemic inflammatory illness, and drug-induced nephritis, nephrology was consulted and a renal biopsy was performed.

The preliminary pathology report, available the same day, demonstrated granulomatous interstitial nephritis with abundant eosinophils. The patient was started on methylprednisolone 125 mg intravenously twice daily, which was continued for four days before transition to oral prednisone 80 mg daily.

Histopathologic examination revealed dense interstitial inflammatory infiltrates composed predominantly of eosinophils and lymphocytes with associated granuloma formation, tubulitis, and interstitial edema, findings most consistent with drug-induced AIN secondary to a hypersensitivity reaction. Representative light microscopy images of the granulomatous interstitial nephritis with eosinophil-rich infiltrates and tubulitis are shown in Figures 2-4.

**Figure 2:**
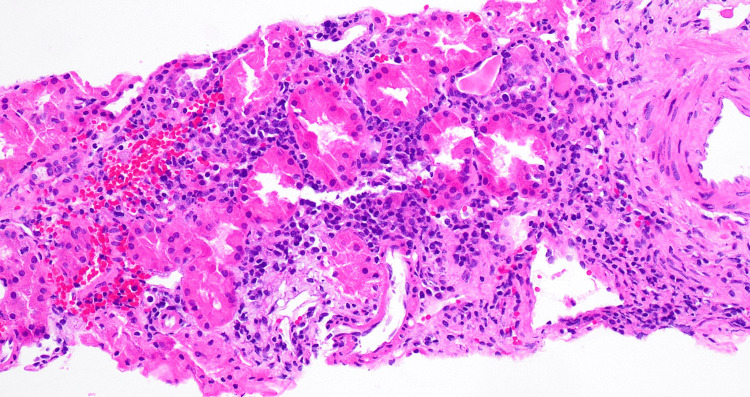
Eosinophil-rich acute interstitial nephritis Renal biopsy demonstrating dense interstitial inflammatory infiltrates composed of lymphocytes, plasma cells, and numerous eosinophils.

**Figure 3:**
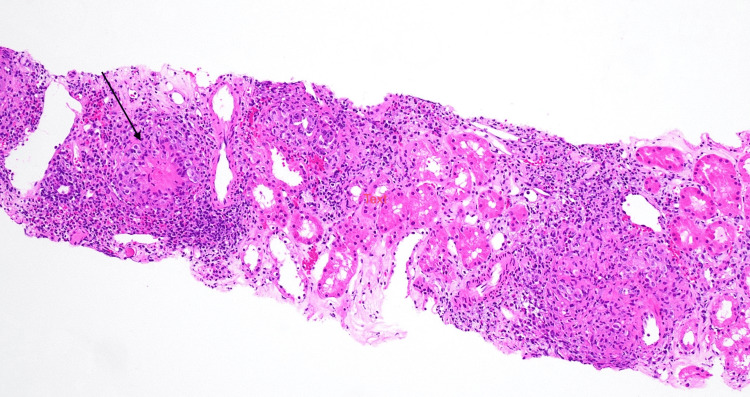
Granulomatous interstitial nephritis Low-power view of renal biopsy demonstrating granulomatous interstitial nephritis with multiple epithelioid granulomas. One granuloma shows a small area of fibrinoid necrosis (arrow).

**Figure 4:**
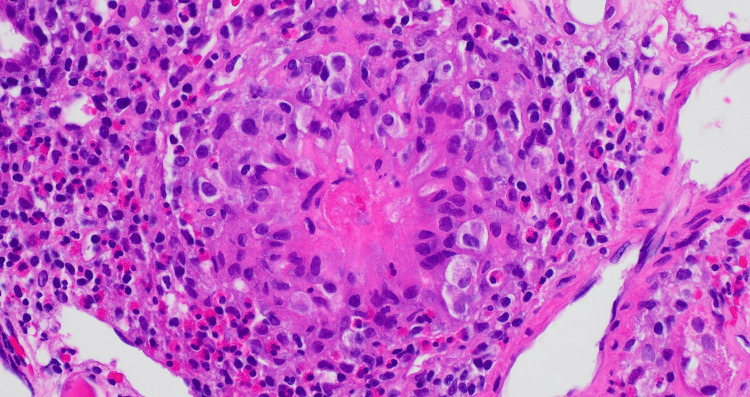
Epithelioid granuloma in drug-induced granulomatous interstitial nephritis High-power view showing an epithelioid granuloma with central fibrinoid change.

Over the ensuing days, his mental status improved, the rash began to desquamate, eosinophilia resolved, and renal function stabilized. His pancreatitis improved with supportive management. He was discharged on hospital day 9 with creatinine improved from a peak of 4.14 mg/dL to 2.7 mg/dL and a plan for a gradual corticosteroid taper.

At two-month follow-up, his creatinine had improved to 1.4 mg/dL, and he remained clinically well.

Table 1 below summarizes the most relevant laboratory values throughout admission.

**Table 1:** Nine‑day laboratory trend ALT, alanine aminotransferase; AST, aspartate aminotransferase.

Parameter	Day 1	Day 2	Day 3	Day 4	Day 5	Day 6	Day 7	Day 8	Day 9	Normal range /units
Creatinine	2.4	2.7	3.4	4.1	3.9	3.8	3.7	3.4	2.7	0.7-1.3 mg/dL
WBC	14.74	16.64	17.64	19.79	21.94	22.14	23.04	24.74	20.24	4.0-9.1 × 10⁹/L
Absolute eosinophils	0.73	1.98	2.73	1.88	1.03	2.65	1.61	3.73	2.63	0.00-0.50 × 10⁹/L
ALT	70	51	67	53.5	40	38	45	40	30	7-52 U/L
AST	42	24	37	26	15	14	13	19	16	13-39 U/L
Lipase	1940	942	772	652	532	398	264	147	30	11-82 U/L

## Discussion

DRESS is a life-threatening hypersensitivity syndrome in which allopurinol accounts for a substantial proportion of cases, particularly in patients with underlying CKD [2]. The two- to six-week latency, progressive morbilliform rash, marked eosinophilia, hepatitis, pancreatitis, and rapidly worsening AKI observed here are classic [1,2]. Renal involvement, most commonly AIN, occurs in up to 40% of patients and carries a higher incidence with allopurinol than with other culprits; the granulomatous eosinophil-rich infiltrate on biopsy strongly supports a drug-induced etiology and represents a less common but recognized variant [3]. Pre-existing CKD heightens susceptibility through impaired clearance of oxypurinol, the active metabolite [2].

Pancreatic involvement in DRESS is uncommon (<5%) but well-reported with allopurinol and should be considered in any patient with abdominal symptoms and lipase elevation; it reflects the systemic inflammatory response rather than direct toxicity [4]. HHV-6 reactivation, documented in this case, occurs in approximately 60% of DRESS patients and is associated with greater disease severity, prolonged course, more flares, and higher risk of multiorgan failure [5].

Altered mental status in this patient likely reflected a multifactorial toxic‑metabolic encephalopathy within the systemic inflammatory milieu of DRESS. Severe AKI, hepatocellular injury, and pancreatitis can each contribute to metabolic derangements that impair cognition, and all were present during the period of neurological decline. HHV‑6 reactivation -- observed in approximately 60% of DRESS cases and associated with greater disease severity -- may further exacerbate neurocognitive dysfunction through heightened systemic inflammation [5]. The rapid improvement in mental status following initiation of high‑dose corticosteroids supports an inflammatory rather than structural etiology and aligns with the expected trajectory of severe DRESS once immunosuppression is started [1,6].

Management centers on immediate withdrawal of the offending agent and systemic corticosteroids for moderate-to-severe organ involvement. High-dose glucocorticoids (prednisone 1 mg/kg/day or equivalent) accelerate resolution of rash, eosinophilia, mental status changes, and organ dysfunction, as evidenced by our patient’s rapid improvement [1,6]. Renal recovery is typical with early therapy, though incomplete recovery or progression to CKD can occur in severe cases [3].

Allopurinol remains first-line urate-lowering therapy for gout, including in CKD, but requires disciplined initiation: start at ≤100 mg/day (≤50 mg/day in CKD stage ≥3) with slow titration to target serum urate while monitoring closely for early hypersensitivity signs in the first two to three weeks [7]. Rapid dose escalation and baseline renal impairment are major risk factors. HLA-B*58:01 screening is conditionally recommended in high-risk ethnic groups (e.g., Southeast Asian and African American) per current guidelines [7]. In this patient, underlying CKD likely amplified risk, underscoring the need for cautious dosing and vigilance.

This case illustrates the pivotal role of renal biopsy in progressive AKI amid competing diagnoses, the supportive diagnostic value of HHV-6 testing, and the importance of prompt corticosteroids in allopurinol-induced DRESS with granulomatous AIN and pancreatitis. Early recognition prevents irreversible organ damage in what remains a diagnostically treacherous syndrome.

## Conclusions

This case highlights a severe presentation of allopurinol‑induced DRESS complicated by granulomatous interstitial nephritis and acute pancreatitis. Early recognition, prompt withdrawal of allopurinol, and initiation of systemic corticosteroids led to rapid clinical improvement and near‑complete renal recovery. Given the widespread use of allopurinol in patients with CKD, clinicians should initiate therapy at low doses, monitor closely for early signs of hypersensitivity, and maintain a high index of suspicion for multiorgan involvement.

In complex multisystem presentations with competing AKI etiologies, renal biopsy plays a pivotal role in clarifying the etiology of AKI and directing timely immunosuppression.
